# An Experimental Study Reveals the Protective Effect of Autophagy against Realgar-Induced Liver Injury via Suppressing ROS-Mediated NLRP3 Inflammasome Pathway

**DOI:** 10.3390/ijms23105697

**Published:** 2022-05-19

**Authors:** Jing Yang, Jian Li, Haoqi Guo, Yuwei Zhang, Ziwei Guo, Yu Liu, Taoguang Huo

**Affiliations:** 1 Department of Health Laboratory Technology, School of Public Health, China Medical University, Shenyang 110122, China; 2019120249@stu.cmu.edu.cn (J.Y.); 2019120254@stu.cmu.edu.cn (J.L.); 2020120316@stu.cmu.edu.cn (H.G.); 2020120337@stu.cmu.edu.cn (Y.Z.); 2021120406@cmu.edu.cn (Z.G.); 2021120404@stu.cmu.edu.cn (Y.L.); 2 Key Laboratory of Arsenic-Related Biological Effects and Prevention and Treatment in Liaoning Province, School of Public Health, China Medical University, Shenyang 110122, China

**Keywords:** realgar, liver injury, autophagy, NLRP3 inflammasome

## Abstract

Realgar, a poisonous traditional Chinese medicine, has been shown to cause liver injury when used for long periods or overdoses. However, the underlying molecular mechanisms and therapeutic targets have not been fully elucidated. The aim of this study is to explore the role of autophagy in sub-chronic realgar exposure-induced liver injury. Here, the liver injury model was established by continuously administrating mice with 1.35 g/kg realgar for 8 weeks. 3-methyladenine (3-MA) and rapamycin (RAPA) were used to regulate autophagy. The results showed that realgar induced abnormal changes in liver function, pathological morphology, expression of inflammatory cytokines, and upregulated NLRP3 inflammasome pathway in mouse livers. RAPA treatment (an inducer of autophagy) significantly improved realgar-induced liver injury and NLRP3 inflammasome activation, while 3-MA (an inhibitor of autophagy) aggravated the realgar-induced liver injury and NLRP3 inflammasome activation. Furthermore, we found that realgar-induced NLRP3 inflammasome activation in mouse livers is mediated by ROS. RAPA eliminates excessive ROS, inhibits NF-κB nuclear translocation and down-regulates the TXNIP/NLRP3 axis, consequently suppressing ROS-mediated NLRP3 inflammasome activation, which may be the underlying mechanism of the protective effect of autophagy on realgar-induced liver injury. In conclusion, the results of this study suggest that autophagy alleviates realgar-induced liver injury by inhibiting ROS-mediated NLRP3 inflammasome activation. Autophagy may represent a therapeutic target in modulating realgar-induced liver injury.

## 1. Introduction

Realgar is a traditional Chinese medicine. In the clinic, it has been used for the treatment of acute promyelocytic leukemia (APL) and other cancers [1]. Realgar is frequently used in combination with herbs to achieve better efficacy and reduce toxicity. In the 2015 edition of the Pharmacopeia of China (Volume 1), approximately 2.48% of the recipes contain realgar among the listed total of 1439 traditional recipes [2]. The major component of realgar is disulfide arsenic (As_2_S_2_) [3]. Although the toxicity of realgar is far less than that of arsenic, increasing evidence supports that the arsenic in realgar can be absorbed into the blood and accumulated in various organs causing systemic damage, predominantly liver, kidney and nerve damage [4]. The liver is the main site of arsenic metabolism as well as the major target of realgar toxicity. More and more studies have reported that the mice exposed to realgar exhibited obvious liver damage [5,6,7]. However, the mechanism of realgar-induced liver injury and the therapeutic target has not been completely elucidated.

The nucleotide-binding oligomerization domain (NOD)-like pyrin domain-containing protein 3 (NLRP3) inflammasome, which consists of NLRP3, adaptor apoptosis-associated speck-like protein (ASC), and pro-caspase-1 is deemed as a vital driving factor of various liver diseases and chemical/toxin-induced liver injury [8]. NLRP3 recruits and cleaves pro-caspase-1 into its active forms, which cleaves pro-interleukin-1β (pro-IL-1β) and interleukin-18 (pro-IL-18) into mature interleukin-1β (IL-1β) and interleukin-18 (IL-18), and finally triggers inflammation occurrence [9]. Furthermore, the active-caspase-1 cleaves gasdermin D (GSDMD) into an N-terminal fragment (N-GSDMD), leading to the formation of membrane pores to mediate pyroptosis. Our recent study has demonstrated that sub-chronic realgar exposure upregulated the NLRP3 inflammasome and promoted the expression and release of IL-1β, leading to inflammatory damage in mouse livers [7].

Autophagy is a process that eliminates damaged organelles and proteins through cytoplasmic degradation [10]. Increasing evidence has revealed that there is a close relationship between autophagy and NLRP3 inflammasome activation [11]. A number of studies have shown that autophagy has a protective effect on exogenous or drugs/toxicant-induced liver injury [12]. However, till now, little is known about the role of autophagy in realgar-induced liver injury and the relationship between autophagy and NLRP3 inflammasome activation in liver tissues of realgar-exposed mice.

Therefore, in this study, 3-methyladenine (3-MA, an inhibitor of autophagy) or rapamycin (RAPA, an inducer of autophagy) was used to regulate autophagy in realgar- induced liver injury mice model to explore the function of autophagy in realgar-induced liver injury and elucidate the relationship between autophagy and NLRP3 inflammasome activation in liver tissues. We aimed to provide insightful viewpoints into the mechanism and therapeutic target of realgar-induced liver injury.

## 2. Material and Methods

### 2.1. Chemical and Reagents

Water-processed realgar was purchased from Sanmenxia Yuhuangshan Pharmaceutical Corporation (Sanmenxia, Henan, China, Lot: W1-180321). N-acetylcysteine (NAC, purity ≥ 98%, #PHR1098) was purchased from Sigma (St. Louis, MO, USA). 3-MA (purity ≥ 99%, #T1879) and Rapa (RAPA, purity ≥ 98%, #T1537) were purchased from TargetMol (Shanghai, China). Alanine aminotransferase (ALT, #c009-2-1), aspartate aminotransferase (AST, #c010-2-1), alkaline phosphatase (ALP, #A059-1-1), total bilirubin (TBIL, #c019-1), lactate lactic dehydrogenase (LDH, #A020-2-2), reactive oxygen species (ROS, #E004-1-1) kits were purchased from Nanjing Jiancheng Bioengineering Institute (Nanjing, China). Assay kits for urea (UN, #R21723-100T) and albumin (ALB, #CEB028Rb) were obtained from Shenyang BoKe Biological Technology Co. Itd and Shenyang Bolande Trading Co., Ltd. (Shenyang, China). Mouse γ- glutamyl transferase (GGT, #JM-11634M2) ELISA Kits were provided by JIANGSU JINGMEI Biotechnology Co., Ltd. (Yancheng, Jiangsu, China). Mouse serum creatinine (Cr, #RX-28589) ELISA kits were purchased from Mode Biological Technology Co. Itd (Shenyang, China). IL-1β (#EK0394) ELISA Array Kits were purchased from Boster Biological Technology Co. Itd (Wuhan, China). Monodansylcadaverine (MDC, #G0170) was purchased from solarbio (Beijing, China). PrimeScript™ RT reagent Kit with gDNA Eraser (#RR820A), TB Green^®^ Premix Ex Taq™ II (RR047A) were purchased from TaKaRa Biotechnology Company (Dalian, China). Antibodies against NLRP3 (#19771-1-AP), Caspase-1 (#22915-1-AP), thioredoxin (TRX, #14999-1-AP), p62 (#18420-1-AP), LC3 (#14600-1-AP), vacuolar protein sorting 34 (VPS34, #12452-1-AP), the mammalian target of rapamycin (mTOR, #66888-1-Ig), nuclear receptor-κB p65 (NF-κB p65, #AF5006) and Lamin B1 (#12987-1-AP) were purchased from Proteintech (Wuhan, China). Antibodies against ASC (#67824) and thioredoxin-interacting protein (TXNIP, #14715) were obtained from Cell Signaling Technology (Beverly, MA, USA). Antibodies against IL-1β (#ab254360), IL-18 (#ab207323) and GSDMD (#ab209845) were obtained from Abcam (Cambridge, UK). Pure Proteome Protein A/G Mix Magnetic Beads (#LSKMAGAG02) were purchased from Merck Millipore (Burlington, MA, USA).

### 2.2. Animals and Treatments

Six-week-old healthy male Kunming (KM) mice (weighing 20~22 g) were obtained from the Laboratory Animal Center of China Medical University (Permission No SCXK2015-0001; Shenyang, Liaoning, China). The experimental mice were housed under SPF conditions under a 12 h light-dark cycle (8:00am–8:00pm) in an air-conditioned room with a room temperature of (25 ± 2) °C and (55 ± 10)% humidity and providing free access to food and water. The experimental protocol was processed according to the National Institute of Health Guide for the Care and Use of laboratory Animals (NIH Publication No.80-23, 1996) and approved by the Animal Ethics Committee of China Medical University. All efforts were exerted to minimize the number of mice used and their suffering.

After one week of adaptive feeding, all mice were randomly divided into the following eight groups: (1) Control group; (2) Realgar group; (3) NAC group; (4) NAC+Realgar group; (5) 3-MA group; (6) 3-MA+Realgar group; (7) RAPA group, and (8) RAPA+Realgar group. Mice in the Control, NAC, 3-MA and RAPA groups received 0.5% CMC-Na; mice in the Realgar, NAC+Realgar, 3-MA+Realgar and RAPA+Realgar groups received 1.35 g/kg realgar by gavage once daily for 8 consecutive weeks. For the intervention studies, NAC, 3-MA, and RAPA were dissolved in 0.5% dimethylsulfoxide (DMSO). Mice in the NAC and NAC+Realgar groups were injected intraperitoneally with NAC at a dose of 20 mg/kg; mice in the RAPA and RAPA+Realgar groups were injected intraperitoneally with RAPA at a dose of 2 mg/kg; mice in the 3-MA and 3-MA+Realgar groups were injected intraperitoneally with 3-MA at a dose of 15 mg/kg. Mice in the Control and Realgar groups were injected intraperitoneally with 0.5% DMSO. All injections were given every two days. NAC was given for eight weeks, while 3-MA and RAPA were given for three weeks before isoflurane anaesthesia.

### 2.3. Measurement of Liver Index and ALT, AST, ALP, γ-GGT, TBIL, ALB, UN, Cr, LDH, IL-1β in Plasma

The liver was removed carefully and rinsed with phosphate buffer. Then, the liver weights were measured after the liver tissues were dried with filter paper to remove the excess solution. The liver index was calculated as follows: liver index% = liver mass (g)/body mass (g) × 100%. The plasma ALT, AST, ALP, γ-GGT, TBIL, ALB, UN, Cr, LDH and IL-1β levels were detected with commercial kits on the basis of the manufacturer’s instructions.

### 2.4. Histopathological Examination

The heart perfusion-fix technique was used to prepare the specimen for histopathological examination. The PBS was perfused initially to clear the blood from the liver followed by 4% paraformaldehyde and 2.5% (*w*/*v*) glutaraldehyde buffer. After perfusion for about 20 min, the liver was excised. Cross-sections (5 µm) of the tissue were cut and stained with hematoxylin and eosin (H&E) and then assessed under a microscope (OLYMPUS, Tokyo, Japan). For transmission electron microscopy (TEM) observation, the liver specimen was cut into 1 mm^3^ in size and fixed in 1% osmium tetroxide. Next, the samples were dehydrated through a graded series of ethanol and acetone and then embedded in the epoxy resin. After being sliced into 1-mm-thick sections and stained with lead citrate, the samples were detected by Hitachi (Ibaraki, Japan) transmission electron microscope.

### 2.5. Evaluation of ROS in Mouse Livers

The intracellular ROS was determined by detecting the fluorescent intensity of 2,7-dichlorodihydrofluorescein diacetate (DCFH-DA). Each liver tissue sample was homogenized in ice-cooled Tris-HCl buffer (40 mM, pH = 7.4, 4 °C). The homogenate 190 μL was mixed with 10 μL DCFH-DA working solution (1 mmol/L), and then incubated in the dark at 37 °C for 30 min. Finally, the fluorescence intensity of DCF was assessed using a FLUOstar Omega^®^ multi-functional microplate reader (Biotek, Winooski, VT, USA) with an excitation wavelength of 502 nm and emission wavelength of 530 nm.

### 2.6. MDC Measurement

The intensity of autophagic vacuoles (AVOs) was measured with MDC. The fresh liver tissue was minced in PBS and digested in 1 mL of trypsin solution at 37 °C for 30 min. Afterwards, 5 mL of DEME was added to terminate digestion. After filtration with sieves, the samples were washed two times using cold PBS and resuspended in binding buffer. The number of viable cells was counted and adjusted to 1 × 10^6^ cells/mL. The single-cell suspension was incubated with MDC (50 μM) for 45 min at room temperature avoiding light, followed by measuring with the help of a microplate reader (Biotek, Winooski, VT, USA) at the excitation wavelength of 335 nm, and the emission wavelength of 518 nm.

### 2.7. RT-qPCR Assay

For RNA extraction, total RNAs from mouse liver tissue were extracted by Trizol Reagent (TaKaRa, Maebashi, Japan) according to the manufacturer’s instructions and the concentration of RNA was calculated. Approximately 1 μg of total RNA from each sample was reverse transcribed to cDNA by the PrimeScript RT reagent kit (TaKaRa, Maebashi, Japan). The levels of mRNA expression were quantified using SYBR Green PCR Master Mix and an ABI prim 7500 Sequence Detection System (Applied Biosystems, Waltham, MA, USA). The PCR program included 95 °C for 5 s and then 95 °C for 30 s and 60 °C for 30 s for 40 cycles. Relative expression of target genes was normalized to *Gapdh*, analyzed by the 2^−ΔΔCt^ method and presented as a ratio compared with the control. The primers of target genes were designed by Sangon Biotech (Shanghai, China) and the sequences are shown in Table 1.

### 2.8. Western Blot

After treatment, RIPA lysis buffer supplemented with protease and phosphatase inhibitors was used to extract the liver protein samples of mice according to the standard procedure. A BCA protein detection kit (Dingguo Changsheng, Beijing, China) was used for the quantification of protein content. After SDS-PAGE electrophoresis, the protein was transferred onto the PVDF membrane. The membranes were blocked using 5% skim milk for 1 h at room temperature. Then, the membrane was hatched by a specific primary antibody at 4 °C overnight. After washing three times with TBST, the membranes were incubated with horseradish peroxidase-conjugated secondary antibodies (Dingguo Changsheng, Beijing, China) for 1 h. The following primary antibodies were used: NLRP3 (1:1000), ASC (1:1000), Caspase-1 (1:1000), IL-1β (1:1000), GSDMD (1:1000), NF-κB (1:1000), TRX (1:1000), TXNIP (1:1000), IL-18 (1:1000), P62 (1:1000), LC3B (1:1500), VPS34 (1:1000), mTOR (1:5000), GAPDH (1:3000), and Lambin B1 (1:2500). GAPDH was used as an invariant control for the target proteins. The expressions of nuclear NF-κB proteins were normalized to Lambin B1 as a reference.

### 2.9. Co-Immunoprecipitation (Co-IP)

In order to detect the protein-protein interactions, a Co-IP assay was performed. RIPA lysis of 1 mL was added to 0.05 g of the liver sample, and then homogenized and centrifuged at 12,000 rpm at 4 °C for 10 min. The supernatant was collected and incubated with mouse monoclonal TXNIP antibody or IgG control antibody for 4 h at 4 °C. Then 20 μL of protein A/G beads (Santa Cruz Biotechnology, Dallas, TX, USA) were added to the protein-antibody complex and incubated for 2 h at 4 °C. Then, the protein-bead mixture was centrifuged and the supernatant was removed. The beads were washed three times with PBST buffer followed by denaturation with 1 × SDS-containing loading buffer at 100 °C for 5 min. Then, the expressions of captured proteins were assessed by Western blot. TXNIP-binding proteins from protein extract were immunoprecipitated with TXNIP antibodies. NLRP3 and TRX levels in pulled-down proteins were measured using Western blot analysis with NLRP3 and TRX antibody.

### 2.10. Statistical Analysis

Data are presented as the mean ± standard error (SEM). IBM SPSS 24.0 software (IBM, Armonk, NY, USA) was used for data analysis. The graphs were made with GraphPad Prism 5.0 software. The Student’s *t*-test was used for statistical analysis of two groups. *p* < 0.05 was considered statistically significant.

## 3. Results

### 3.1. Autophagy in Realgar-Exposed Mouse Livers

**Figure 1:**
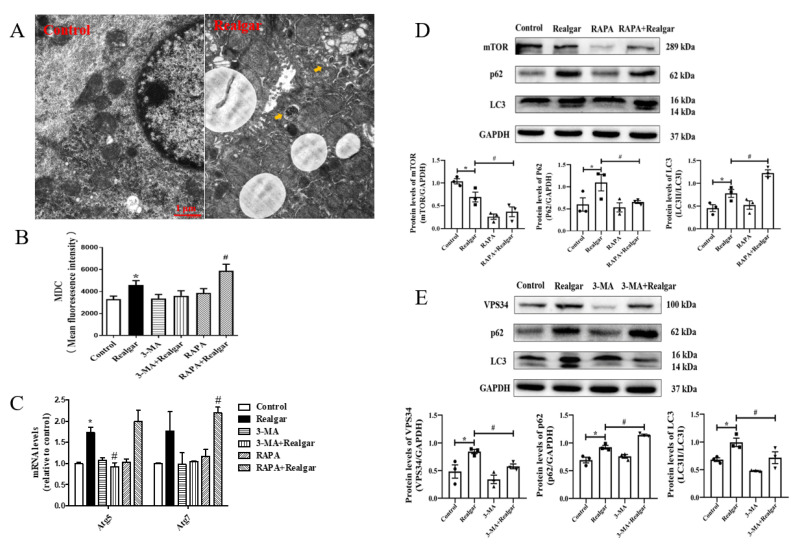
Regulation of autophagy in mouse livers. (**A**) Electron microphotographs showed the changes of hepatocytes. scale bar:1μm; magnified 20,000×. The arrows refer to the autophagic vacuoles. (**B**) The fluorescence intensity of MDC-labeled autophagic vacuoles (*n* = 6). (**C**) The relative mRNA levels of *Atg5* and *Atg7* were evaluated by RT-qPCR and normalized the data to *Gapdh* (*n* = 3). (**D**) Representative Western blots and quantitative analysis of the protein expressions of mTOR, P62 and LC3II/I in RAPA treated groups. The gray value of the bands was normalized to GAPDH (*n* = 3). (**E**) Representative Western blots and quantitative analysis of the protein expressions of VPS34, P62 and LC3II/I in 3-MA treated groups. The gray value of the bands was normalized to GAPDH (*n* = 3). Data are presented as mean ± SEM. * *p* < 0.05, compared with control group; # *p* < 0.05, compared with realgar-treated group.

We first measured autophagy in realgar-exposed mouse livers. Increased numbers of autophagosomes were observed in the Realgar group under the TEM (Figure 1A). The intensity of MDC fluorescence was remarkably elevated in the Realgar group (Figure 1B). Western blot and RT-qPCR were used to measure the expression of autophagy-related proteins and genes. Increased expressions of *Atg5* mRNA and VPS34, LC3II/I, and P62 proteins were observed in the Realgar group as compared with controls (Figure 1C,D). The results suggested that realgar caused autophagy dysregulation in mouse livers.

In order to investigate the role of autophagy in realgar-induced liver injury, RAPA and 3-MA, the classical autophagic agonist and antagonist, were used to regulate the autophagy in vivo [13]. In comparison to the Realgar group, the intensity of MDC fluorescence in the RAPA+Realgar group was apparently increased (Figure 1B); the mRNA expression of *Atg7* was increased (Figure 1C, *p* < 0.05), and the protein expressions mTOR and P62 were significantly lower, while the ratio of LC3II and LC3I was distinctly higher than that of the Realgar group (Figure 1D, *p* < 0.05). In mice treated with 3-MA+Realgar, opposite results were obtained from the RAPA+Realgar group. The mRNA expression of *Atg5* was decreased (Figure 1C, *p* < 0.05), and the protein expression of VPS34, the ratio of LC3II and LC3I were down-regulated, whereas the protein expression of P62 was up-regulated (Figure 1E, *p* < 0.05) as compared with the Realgar group. These results have demonstrated that RAPA and 3-MA can efficiently regulate autophagy in realgar-exposed mouse livers.

### 3.2. Autophagy Alleviates Realgar Induced Damages in Mouse Livers

**Figure 2:**
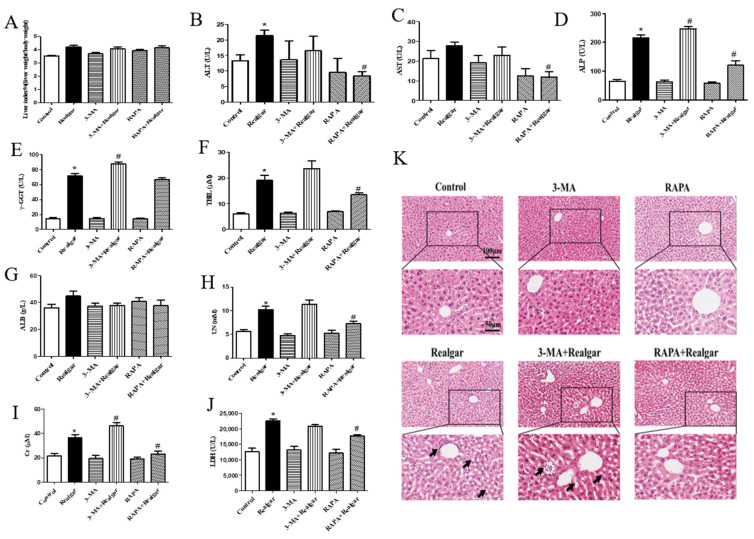
The effect of autophagy on realgar-induced liver injury. The liver index (**A**). The ALT (**B**) and AST (**C**), ALP (**D**), γ-GGT (**E**), TBIL (**F**), ALB (**G**), UN (**H**), Cr (**I**) and LDH (**J**) levels in mouse plasma (*n* = 6). Data are presented as mean±SEM. * *p* < 0.05, compared with control group; # *p* < 0.05, compared with realgar-treated group. (**K**) Representative images of morphological changes in mouse liver examined by H&E staining (200×, 400×). The scale is 100 μm and 50 μm. The arrows refer to cytoplasm vacuolization, focal nuclear pyknosis or inflammatory infiltration.

Biochemistry measurement and H&E staining were used to evaluate the effect of autophagy on realgar-induced damages in mouse livers. Compared with the control group, the liver index of mice in each group did not apparently change (Figure 2A). As shown in Figure 2B–J, the plasma ALT, ALP, γ-GGT activities and TBIL, UN, Cr, and LDH levels in realgar-exposed mice were 1.6-, 3.3-, 4.94-, 3.19-, 1.82-, 1.69- and 1.79-times higher than those of the control mice, respectively (*p* < 0.05), and the plasma AST activity of mice exposed to realgar showed an increasing trend (*p* > 0.05). The ALT, AST, ALP activities and TBIL, UN, Cr, and LDH levels in the plasma of mice in the RAPA+Realgar group were significantly lower than those of the Realgar group (ALT: 60.9%; AST: 55.2%; ALP: 55.7%; TBIL: 70.4%; UN: 70.6%; Cr: 63.4%, LDH: 21.3% reduction in the Realgar group, *p* < 0.05). The ALP, γ-GGT activities and TBIL, Cr levels in the plasma of mice in the 3-MA+Realgar group were further increased in comparison to the Realgar group (ALP: 1.15 times; γ-GGT: 1.22 times; TBIL: 1.23 times; Cr: 1.26 times of the Realgar group, *p* < 0.05). The level of ALB in mouse plasma was not obviously changed in any group. The results of H&E staining showed that the morphology of hepatocytes in the Realgar group revealed obvious damage (Figure 2E), including the number of normal cells decreased and some cells showed cytoplasm vacuolization, focal nuclear pyknosis and inflammatory infiltration. However, the morphology of hepatocytes in mouse livers was obviously improved in the Realgar+RAPA group, while there was more serious damage in the 3-MA+Realgar group. These findings together suggest that realgar-induced liver damage was alleviated by autophagy enhancement.

### 3.3. Autophagy Reduces Pro-Inflammatory Cytokines Expression and Release Induced by Realgar

**Figure 3:**
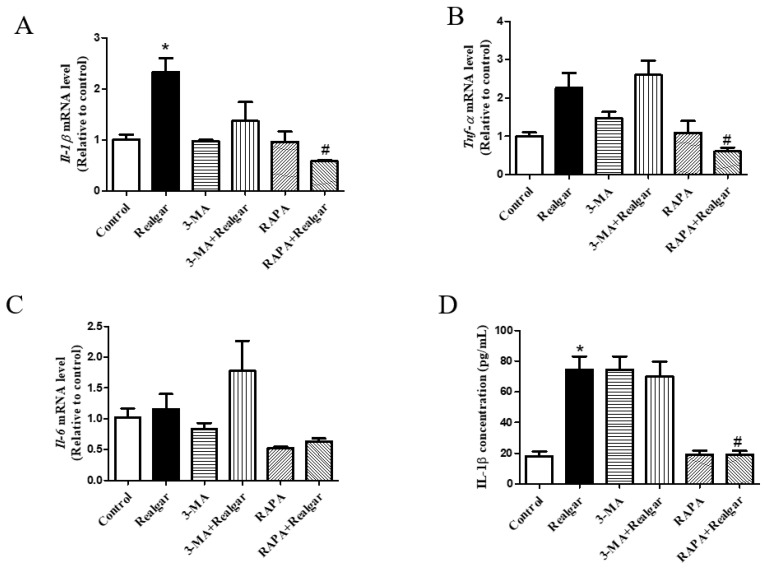
The effect of autophagy on pro-inflammatory cytokines expression and release. (**A**–**C**) The relative mRNA levels of *Il-1β*, *Tnf-a*, *Il-6* were evaluated by RT-qPCR and normalized the data to *Gapdh* (*n* = 3). (**D**) The IL-1β level in mouse plasma (*n* = 6). Data are presented as mean ± SEM. * *p* < 0.05, compared with control group; # *p* < 0.05, compared with realgar-treated group.

The mRNA expression of *Il-1β* in liver tissue of mice exposed to realgar was 2.31 times higher than that of the control group, and the difference was statistically significant (Figure 3A, *p* < 0.05). Compared with the control group, the mRNA expression of *Tnf-α* in the liver tissue of realgar exposed mice tended to increase (Figure 3B). However, there was no significant difference in mRNA expression of *Il-6* in liver tissues between the control and the Realgar group (Figure 3C). The expression of *Il-1β* and *Tnf-α* declined significantly after RAPA (74.7% and 73.7% reduction in realgar group) treatment (Figure 3A,B, *p* < 0.05).

The plasma IL-1β level in realgar exposed mice was 4.09 times higher than those of controls, while the IL-1β level apparently dropped in the RAPA+Realgar group (43% reduction in realgar group) (Figure 3D, *p* < 0.05).

### 3.4. Autophagy Retard Realgar Induced NLRP3 Inflammasome Activation in Mouse Livers

**Figure 4:**
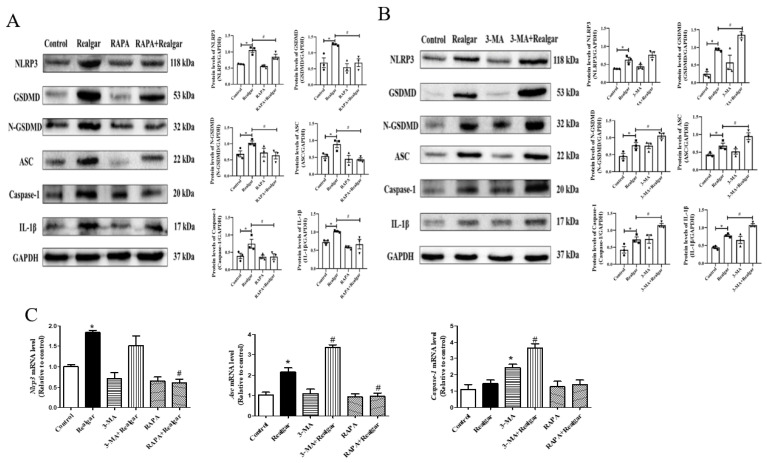
Autophagy retard realgar induced NLRP3 inflammasome activation in mouse livers. (**A**) Representative Western blots and quantitative analysis of the expressions of NLRP3 inflammasome-related proteins in RAPA treated groups. The gray value of the bands was normalized to GAPDH (*n* = 3). (**B**) Representative Western blots and quantitative analysis of the expressions of NLRP3 inflammasome-related proteins in 3-MA treated groups. The gray value of the bands was normalized to GAPDH (*n* = 3). (**C**) The relative mRNA levels of *Nlrp3*, *Asc* and *Caspase-1* were evaluated by RT-qPCR and normalized the data to *Gapdh* (*n* = 3). Data are presented as mean ± SEM. * *p* < 0.05, compared with control group; # *p* < 0.05, compared with realgar-treated group.

The protein expressions of NLRP3, ASC, Caspase-1, IL-1β, GSDMD, N-GSDMD and mRNA expressions of *Nlrp3*, *Asc* were all distinctly higher in realgar-exposed mouse livers than those in controls (Figure 4A–C), which is consistent with our previous result [7]. Compared with the Realgar group, the protein expression of NLRP3, ASC, Caspase-1, IL-1β, GSDMD, N-GSDMD (Figure 4A) and the mRNA expression of *Nlrp3*, *Asc* (Figure 4C) were all significantly decreased in the RAPA+Realgar group (*p* < 0.05), which suggesting that autophagy enhancement can retard realgar induced NLRP3 inflammasome activation in mouse livers. The changes of NLRP3 inflammasome-related proteins and genes in the 3-MA+Realgar group were opposite to those of the RAPA+Realgar group. The protein expressions of ASC, Caspase-1, IL-1β, GSDMD, N-GSDMD (Figure 4B) and the mRNA expressions of *Asc*, *Caspase-1* (Figure 4C) were remarkably increased (*p* < 0.05) in the 3-MA+Realgar treatment group, suggesting that inhibition of autophagy aggravated NLRP3 inflammasome activation induced by realgar.

### 3.5. ROS Production Contributes to Realgar-Induced NLRP3 Inflammasome Activation in Mice Livers

**Figure 5:**
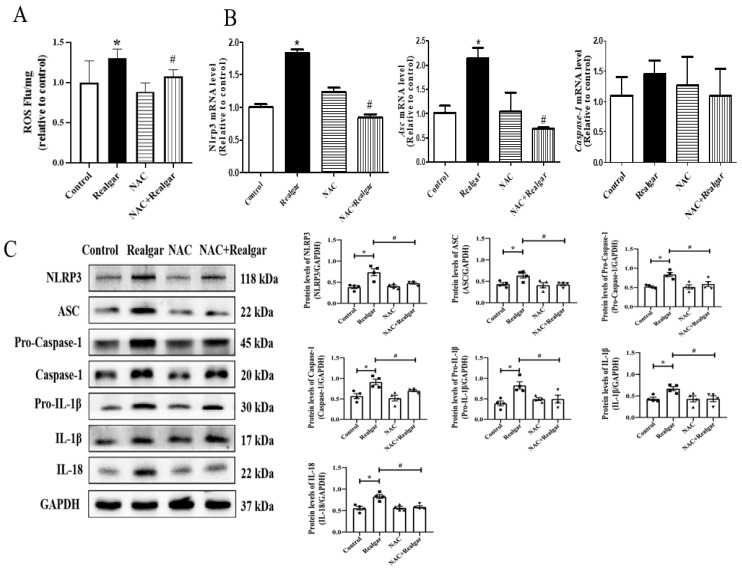
ROS is involved in realgar-induced NLRP3 inflammasome activation in mouse livers. (**A**) The intracellular ROS level in mouse livers was measured by the DCFH-DA method (Ex 502 nm, Em 530 nm, *n* = 6). (**B**) The relative mRNA levels of *Nlrp3*, *Asc*, *Caspase-1* were evaluated by RT-qPCR and normalized the data to *Gapdh* (*n* = 3). (**C**) Representative Western blots showing the expressions of NLRP3 inflammasome-related proteins and quantitative analysis using image J software. The gray value of the bands was normalized to GAPDH (*n* = 4).Data are presented as mean ± SEM. * *p* < 0.05, compared with control group; # *p* < 0.05, compared with realgar-treated group.

As shown in Figure 5A, realgar exposure markedly elevated the ROS level in mouse livers, which is consistent with our previous report [7]. When pretreated with NAC, an ROS scavenger, the changes in NLRP3 inflammasome-related genes and proteins induced by realgar exposure were obviously restored (Figure 5B,C). These findings confirm the role of ROS in NLRP3 inflammasome activation by realgar exposure.

### 3.6. Autophagy Eliminates Excessive ROS, Inhibits NF-κB Nuclear Translocation and Down-Regulates TXNIP/NLRP3 Axis in Realgar Exposed Mouse Livers

**Figure 6:**
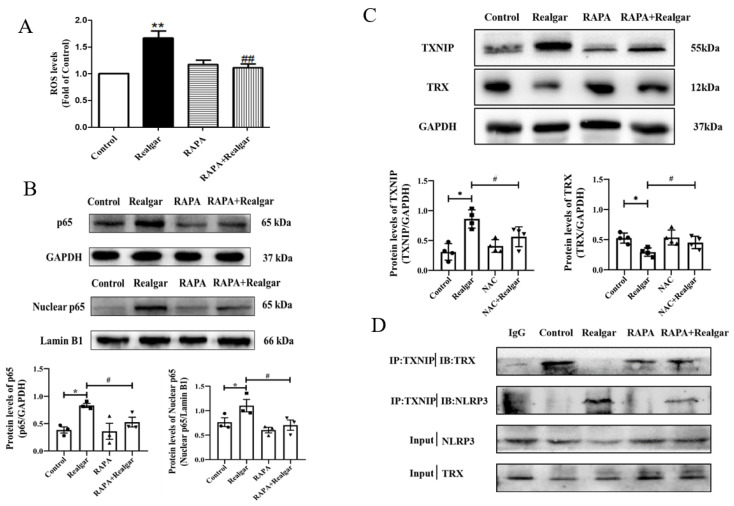
Autophagy eliminates excessive ROS, inhibits NF-κBp65 nuclear translocation and down-regulated TXNIP/NLRP3 axis in realgar exposed mouse livers. (**A**) The effect of RAPA on ROS level in realgar-exposed mouse livers (*n* = 6). (**B**) Representative Western blots and quantitative analysis of the expressions of total NF-κB p65 and Nuclear-NF-κB p65 in RAPA treated groups. The gray value of the bands was normalized to GAPDH (*n* = 3). (**C**) Representative Western blots and quantitative analysis of the expressions of TXNIP and TRX in RAPA treated groups. The gray value of the bands was normalized to GAPDH (*n* = 3). (**D**) Immunoprecipitation and immunoblot analysis of the interaction between TXNIP and TRX, TXNIP and NLRP3 in mouse livers in RAPA treated groups. Data are presented as mean±SEM. * *p* < 0.05, ** *p* < 0.01, compared with control group; # *p* < 0.05, ## *p* < 0.01, compared with realgar-treated group.

Several studies reveal that autophagy can indirectly suppress inflammasome activation by removing ROS [14]. In this experiment, the ROS level in the livers of mice treated with RAPA+Realgar significantly decreased compared with that in the Realgar group (Figure 6A, *p* < 0.05), which indicates that autophagy can eliminate realgar-induced excessive ROS. 

NF-κB p65 is a vital factor in the initiation of NLRP3 inflammasome activation. Compared with the control group, the total and nuclear protein expression NF-κBp65 in mouse livers were significantly increased (Figure 6B, *p* < 0.05). Compared with the Realgar group, the expressions of NF-κBp65 in liver tissues and nuclear in the RAPA+Realgar group were significantly decreased (Figure 6B, *p* < 0.05). The result suggested that enhanced autophagy can inhibit the entry of NF-κBp65 into the nucleus.

More and more evidence supports that ROS-mediated NLRP3 inflammasome activation is linked with TXNIP/NLRP3 axis. Under high ROS concentration, TXNIP disassociates from TRX, and directly interacts with NLRP3, activating the NLRP3 inflammasome [15]. In this experiment, we found that autophagy enhancement decreased the protein expression of TXNIP and increased protein expression of TRX (Figure 6C, *p* < 0.05), and reduced interaction between TXNIP and NLRP3 in realgar-induced liver injury mice (Figure 6D). The findings indicated that autophagy enhancement down-regulates TXNIP/NLRP3 axis.

**Figure 7:**
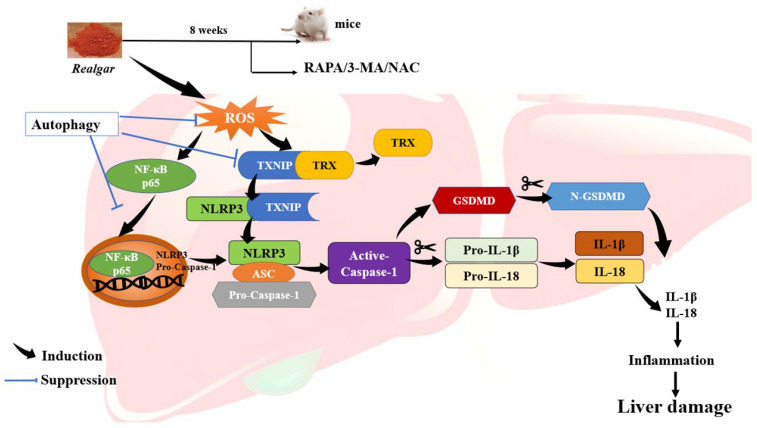
Proposed mechanism of the protective effect of autophagy on realgar-induced liver injury. Autophagy can alleviate realgar-induced liver injury via eliminating excessive ROS, inhibiting NF-κB nuclear translocation and down-regulating TXNIP/NLRP3 axis, consequently inhibiting ROS-mediated NLRP3 inflammasome activation.

Overall, our findings suggest that autophagy eliminates excessive ROS, inhibits NF-κBp65 nuclear translocation and down-regulates the TXNIP/NLRP3 axis in realgar-exposed mouse livers, which may contribute to the inhibitory effect of autophagy on NLRP3 inflammasome activation (Figure 7).

## 4. Discussion

Arsenic is a highly toxic substance, which has been classified as a class I carcinogen by the International Agency for Research on Cancer (IARC). As the main organ of arsenic metabolism and accumulation, the liver is easily affected by arsenic toxicity. Epidemiology studies have indicated a close link between chronic arsenic exposure and abnormal liver function, hepatomegaly, hepatoportal sclerosis, liver fibrosis, cirrhosis and liver cancer in humans [16]. Medicinal exposure is one of the sources of arsenic exposure in humans. Arsenic trioxide (ATO) is a valuable drug in traditional Chinese medicine (TCM), which is often used to treat APL and other cancers [17]. There is a report that for long-term use (>1 month), ATO induced liver damage in about 7.9% of cases [18].

Many herbal medicines also contain arsenic, such as Salviae Miltiorrhizae, Safflower, Gentiana, Flower of Silktree Albizzia, etc. [19]. Realgar is the most famous arsenic-containing drug, which is the essential component of some popular medicinal preparations in Asian and Western countries for the treatment of APL [20]. Realgar has been widely used in diverse formulae in traditional Chinese medicine (TCM), such as *Niuhuang Jiedu* tablet, *Angong Niuhuang* Wan, etc. These realgar-containing TCMs have shown beneficial effects on detoxication, insecticidal, dampness elimination and dispelling phlegm [1]. Studies have shown that realgar has multi-system toxicity [4]. Excessive or long-term exposure to realgar has detrimental influences on the liver [4]. Autophagy is a process of “self-digestion” in which proteins, organelles and other cellular components damaged are transported to lysosomes for degradation. There is evidence that autophagy has a protective effect on drug-induced liver injury [21]. To date, the direct effect of autophagy on realgar-induced liver injury has not been studied.

In previous studies, we found that mouse exposure to realgar at 1.35 g/kg for 8 weeks exhibits obvious liver injury [5,7,22,23]. Herein, mice were given 1.35 g/kg realgar for 8 weeks to induce liver injury. We first examined the effect of realgar exposure on autophagy in mouse livers. TEM is the most classical method for observing autophagic vacuoles. Under TEM, autophagosomes have a double membrane containing morphologically intact cytoplasm, including ribosomes and rough ER, and the limiting membrane that is partially visible as two bilayers separated by a narrow electron-lucent cleft [24]. Autolysosomes typically have only one limiting membrane, which usually contains electron-dense cytoplasmic material and/or organelles at various stages of degradation [25]. In the experiment, an increase in the numbers of autophagic vacuoles in mouse livers after realgar exposure was observed (shown in Figure 1A). MDC, which can accumulate as a selective fluorescent marker for autophagic vacuoles under in vivo conditions by interacting with membrane lipids that are highly concentrated in the autophagic compartments [26], is widely used to detect autophagic vacuoles. In our experiment, the intensity of MDC fluorescence was remarkably elevated in the realgar group as compared with controls (Figure 1B), which further confirmed that realgar led to increased autophagic vacuoles accumulation in mouse livers. In addition, we further detected autophagy-related markers to determine the effect of realgar on autophagy. Vacuolar protein sorting 34 (VPS34) plays an essential role in phagophores nucleation and elongation [27]. In the nucleation process, VPS34 forms a multiprotein complex with Beclin-1. It was found that realgar markedly upregulated the protein expression of VPS34 in mouse livers (Figure 1E). Atg5 and Atg7 are involved in the expansion of the phagophore. The Atg5 conjugated by Atg12 is present on the outer side of phagophores and essential for elongation [28]. Under stress conditions, Atg proteins are activated, resulting in the formation and maturation of autophagosomes from phagophores. In our study, we found that realgar upregulated Atg5 and Atg7 mRNA levels (Figure 1C), suggesting that realgar promotes phagophores elongation. LC3, a marker of the autophagosome, is conversed from LC3I to LC3II when autophagy is induced. Therefore, the ratio of LC3II and LC3I is used as a sign of autophagy induction [29]. In the realgar-exposed mouse livers, the ratio of LC3II to LC3I is remarkably elevated in comparison to that in the control mice (Figure 1D,E), suggesting an induction of autophagy by realgar treatment. P62 serves as an autophagy receptor protein, which can selectively recognize autophagic cargo and mediate its engulfment into autophagosomes to initiate the degradation [30]. P62 accumulates when autophagy is inhibited, and the level of P62 decreases when autophagy is induced. In this study, a significantly upregulated expression of P62 was found in realgar exposed mouse livers (Figure 1D,E), indicating that the autophagy-lysosome pathway was inhibited and autophagy dysfunction occurred in the liver of mice exposed to realgar. This is consistent with the increased numbers of autophagic vacuoles in realgar exposed mouse livers observed by electron microscopy. Similar results were found in the cerebral cortex of realgar exposed rats, which suggests that realgar exposure promotes the initiation of autophagy and the bilayer membrane structure of autophagosome, but blocks autophagy degradation functions [31]. It is well known that autophagy can be induced under stressful conditions, such as oxidative stress, and plays an important role in the modulation of cell death and survival. It has been reported in our previous study that the level of GSH and the activities of GSH-Px, SOD were significantly decreased and the level of ROS markedly increased in the liver tissues of mice treated with the same realgar dose and exposure time as this study [7,23]. Therefore, it is conceivable that autophagy enhancement in realgar-exposed mouse livers is likely a stress-protective response under extensive oxidative stress induced by realgar [5].

RAPA and 3-MA were used to regulate the level of autophagy in the livers of realgar-exposed mice. 3-MA is the most commonly used autophagy inhibitor, and it functions by inhibiting VPS34. There is a report that pointed out that the generation of phosphatidyl-inositol-3-phosphate (PI3P) by VPS34 and the recruitment of PI3P-binding proteins is a critical step for phagophores nucleation and elongation [27]. Therefore, 3-MA inhibited autophagy in an early stage. In this experiment, we found that 3-MA significantly inhibited the levels of Atg5 mRNA and VPS34, LC3II/I proteins, while upregulating the expression of P62 in realgar-exposed mouse livers (Figure 1C–E), suggesting that 3-MA inhibits autophagy in realgar exposed mouse livers. RAPA is an inhibitor of the mammalian target of rapamycin (mTOR), which can induce autophagy by inhibiting the mTOR signaling pathway. After RAPA treatment, the intensity of MDC fluorescence, Atg7 mRNA and LC3II/I ratio were further increased in realgar exposed mouse livers, while the expression of P62 was decreased (Figure 1C–E). The results showed that RAPA could significantly enhance autophagy in the liver tissues of mice from the Realgar group.

Next, we investigated the effect of autophagy on realgar-induced liver injury. It was found that after 8-week realgar exposure, the plasma ALT, ALP, γ-GGT activities and TBIL, UN, Cr, LDH levels are all apparently increased. In particular, the ALP, γ-GGT activities and TBIL levels are far higher than those in the controls, which may indicate that realgar may induce cholestatic liver injury in mouse livers [32]. The results are consistent with our previous study that reported a disturbance in bile acid synthesis and metabolism in liver tissues of mice treated with the same realgar dose and exposure time as this study [7]. RAPA and 3-MA intervention significantly altered the liver functions of realgar-exposed mice. It was found that RAPA markedly restored the elevated levels of ALT, AST, ALP, TBIL, UN, Cr and LDH in mouse plasma induced by realgar, while 3-MA aggravates realgar-induced liver function disorder by further increasing the levels of ALP, γ-GGT, TBIL and Cr in plasma of realgar exposed mice. H&E staining results also suggested that RAPA mitigated realgar-induced liver injury, whereas 3-MA exacerbated. Inflammation is regarded as one of the major mechanisms of realgar-induced liver injury. NLRP3 inflammasome plays an important role in initiating and sustaining inflammation [33]. A previous study has reported that realgar-induced liver injury in mice is related to the activation of the NLRP3 inflammasome pathway [7]. Autophagy is closely related to the activation of NLRP3 inflammasome [11]. The effect of autophagy on NLRP3 inflammasome activation induced by realgar has not been investigated. In this study, we found that autophagy enhancement by RAPA treatment mitigated realgar-induced NLRP3 inflammasome activation in mouse livers and reduced the expression and release of inflammatory cytokines. Oppositely, autophagy inhibition by 3-MA treatment aggravated realgar-induced NLRP3 inflammasome activation in mouse livers and promoted the expression and release of inflammatory cytokines. These results suggest that the ameliorative effect of autophagy on realgar-induced liver injury may be attributed to the inhibition of NLRP3 inflammasome activation.

To elucidate the mechanism by which autophagy inhibits the activation of NLRP3 inflammasome in realgar-induced liver injury mice, we focus on the role of ROS. As we know, ROS is one of the major endogenous danger-associated molecular patterns (DAMPs) that leads to the activation of NLRP3 [34]. In order to clarify whether ROS production is an upstream mechanism for NLRP3 activation induced by realgar, we used NAC, an ROS scavenger, to intervene. The results illustrated that NAC intervention obviously inhibited NLRP3 inflammasome activation in realgar-exposed mouse liver, which suggests that realgar-induced NLRP3 inflammasome activation in mice liver is mediated by ROS.

Autophagy may inhibit NLRP3 inflammasome activation by removing excess ROS. In this experiment, RAPA treatment apparently restored the increased ROS levels induced by realgar in mouse livers.

NF-κB plays an important role in the priming stage of NLRP3 inflammasome activation. The NF-κB enters into the nuclear and upregulates the expression of inflammasome component NLRP3 and pro-IL-1β. It has been reported that autophagy can directly regulate NF-κB to regulate inflammatory responses [35]. Our previous study has shown that sub-chronic realgar exposure activated the NF-κB signaling pathway [7]. In this study, we investigated the effect of autophagy on the NF-κB signaling pathway in realgar-induced liver injury mice. The results showed that RAPA treatment inhibits NF-κBp65 nuclear translocation and subsequently reduced the transcription of *Nlrp3*, *Asc* and *Il-1β*. Therefore, we hypothesized that autophagy may down-regulate the realgar-induced NLRP3 inflammasome activation pathway by inhibiting NF-κB nuclear translocation, thereby alleviating realgar-induced inflammatory injury in mouse livers.

Growing evidence has shown that TXNIP is an active regulator of NLRP3 [36]. It can directly interact with NLRP3 leading to the activation of NLRP3 inflammasome [37]. Heo et al. demonstrated that hepatocytes treated with ethanol resulted in TXNIP overexpression, activating NLRP3 inflammasome [38]. In a previous study, it was found that the gene and protein expression of TXNIP was increased in the liver tissues of mice exposed to 1.35 g/kg realgar for 8 weeks, and the interaction between NLRP3 and TXNIP was enhanced [7]. The TXNIP/NLRP3 axis is regulated by ROS levels. Under high ROS concentrations, TXNIP dissociates from TRX, binds to NLRP3 protein, and activates the NLRP3 inflammasome. We found that activation of autophagy reduced TXNIP protein levels in the liver tissues of realgar-induced liver injury mice and weakened the interaction between TXNIP and NLRP3.

Taken together, this study suggests that autophagy may exert a protective effect on realgar-induced liver injury by eliminating excessive ROS, inhibiting NF-κB nuclear translocation and down-regulating TXNIP/NLRP3 axis, consequently inhibiting NLRP3 inflammasome activation in mouse livers.

## 5. Conclusions

In conclusion, in this study, we demonstrated that autophagy can alleviate realgar-induced liver injury via eliminating excessive ROS, inhibiting NF-κB nuclear translocation and down-regulating the TXNIP/NLRP3 axis, consequently inhibiting ROS-mediated NLRP3 inflammasome activation. The regulation of autophagy would be a potential strategy for preventing and curing realgar-induced liver toxicity, which will be worthy of further study.

## 6. Limitations of the Report

This report has some limitations. First, the RNA and proteins were extracted from whole livers in this experiment, thus, the analysis cannot discriminate between toxicity effects happening in hepatocytes versus other liver cells. In future studies, purified hepatocytes from the livers should be used to avoid confounding effects resulting from other cell types. Second, the suggested autophagy may exert a protective effect on realgar-induced liver injury via down-regulating TXNIP/NLRP3 axis, which needs confirmation by knocking down TXNIP.

## Figures and Tables

**Table 1:** PCR Primer Sequences.

Gene	Forward Primer	Reverse Primer
*Nlrp3*	GCCGTCTACGTCTTCTTCCTTTCC	CATCCGCAGCCAGTGAACAGAG
*Il-6*	CTCCCAACAGACCTGTCTATAC	CCATTGCACAACTCTTTTCTCA
*Tnf-α*	CAGGCGGTGCCTATGTCTC	CGATCACCCCGAAGTTCAGTAG
*Il-1β*	GAAATGCCACCTTTTGACAGTG	TGGATGCTCTCATCAGGACAG
*Asc*	GACAGTGCAACTGCGAGAAG	CGACTCCAGATAGTAGCTGACAA
*Caspase-1*	ACAAGGCACGGGACCTATG	TCCCAGTCAGTCCTGGAAATG
*Atg5*	TGTGCTTCGAGATGTGTGGTT	GTCAAATAGCTGACTCTTGGCAA
*Atg7*	GTTCGCCCCCTTTAATAGTGC	TGAACTCCAACGTCAAGCGG
*Gapdh*	AGGTCGGTGTGAACGGATTTG	GGGGTCGTTGATGGCAACA

## Data Availability

All data generated or analyzed during this study are included in this published article.

## Abbreviations

3-MA: 3-methyladenine; ALT: alanine aminotransferase; ASC: apoptosis-associated speck-like protein containing CARD; AST: aspartate aminotransferase; AVOs: autophagic vacuoles; DCFH-DA: 2,7-dichlorodihydrofluorescein diacetate; DMSO: dimethylsulfoxide; IL-1β: interleukin-1β: LDH: lactate lactic dehydrogenase; MDC: monodansylcadaverine; mTOR: the mammalian target of rapamycin; NAC: N-acetylcysteine; NLRP3: pyrin domain containing protein 3; pro-IL-1β: pro-interleukin-1β; RAPA: rapamycin; ROS: reactive oxygen species; TRX: thioredoxin; TXNIP: thioredoxin-interacting protein; VPS34: vacuolar protein sorting 34.
